# Efficacy and safety of COVID-19 vaccines for patients with spinal tumors receiving denosumab treatment: An initial real−clinical experience study

**DOI:** 10.3389/fonc.2023.1034466

**Published:** 2023-03-22

**Authors:** Pengru Wang, Bo Li, Shangbin Zhou, Yingye Xin, Zhipeng Zhu, Shujie Duan, Danyang Bai, Hao Yuan, Wei Xu, Jianru Xiao

**Affiliations:** ^1^ Department of Orthopedic Oncology, Changzheng Hospital, Second Military Medical University, Shanghai, China; ^2^ Naval Medical Center, Naval Military Medical University, Shanghai, China; ^3^ Department of Orthopedic, Changning County People's Hospital, Yunnan, China

**Keywords:** COVID-19, vaccine, spinal tumor, denosumab, immunology

## Abstract

**Background:**

Even if COVID-19 vaccine has gradually been adopted in the world, information of side effects and crosstalk in patients with spinal tumors is absent due to the exclusion from clinical research. In this research, we aimed to investigate the efficacy and safety for the patients with spinal tumors treated by denosumab.

**Methods:**

In this retrospective research, 400 patients under treatment of denosumab against spinal tumors in real-clinical experience were grouped into two cohorts according to the treatment of COVID-19 vaccine. And linked hospital data, serum samples and unsolicited related adverse events had been collected from January 22nd 2021 to June 1st 2021 respectively.

**Results:**

233 patients of all participants who received regular treatment of denosumab were vaccinated by mRNA or inactivated vaccine. Patients of metastatic disease and primary osseous spinal tumor showed similar distribution in both two groups. Over the study period, within 176 patients tested the status of serologic response of vaccine, 88(81.48%) and 41(87.23%) individuals injected one or two inactivated vaccines had effective antibody against SARS-CoV-2 infections. As 21 patients (85.71%) treated by mRNA vaccine did. Considering of the safety of vaccine, most common systemic adverse events were nausea or vomiting (45 events vs 23events). Interestingly, fewer participants in the vaccine group were statistically recorded in local adverse events than in the placebo group (16 events vs 33 events).

**Conclusions:**

Our initial real-clinical experience suggests that COVID-19 vaccines are likely safe and effective in in patients with spinal tumors receiving denosumab treatment.

## 1 Introduction

The sudden explosion and pandemic caused by coronavirus disease 2019 (COVID-19) (1), which belongs to the same RNA coronavirus family as SARS-CoV and MERS-CoV (2), appears to have been unprecedented. This pandemic has already caused great challenges for the global public health system and has left a heavy burden on the global economy (3). Since the prevalence of cancer seems to be associated with a higher mortality rate due to COVID-19, the development of safe and effective treatments may be crucial for this group of patients (4). Even if several therapies have been tested, including the use of antivirals, corticosteroids, and respiratory support, no specific drug treatments have proven effective against COVID-19 (5). Vaccination is still regarded as the most promising intervention against the spread of the virus. Most patients with spinal tumors are in a generally poor condition or a systemic immunosuppressive state, which can lead to a higher-risk of severe disease and death (6). Furthermore, once these patients have been infected by COVID-19, fatal outcomes may lead to delays in further treatment and a further decline in quality of life (7). However, no studies have focused on the efficacy and safety of COVID-19 vaccines for patients with spinal tumors.

Denosumab, a human monoclonal antibody that targets human RANKL, has been widely used for patients with primary and metastatic spinal tumors. RANKL and RANK are also expressed by cells in the immune system apart from osseous tissue. Meanwhile, RANKL and RANK also played an important role in various immune processes, including lymph node development and activation of dendritic cells, monocytes, and T cells (8–11). Due to the inhibition of the RANK pathway, denosumab may theoretically increase the risk of infections. In the Fracture Reduction Evaluation of Denosumab in Osteoporosis Every 6 months (FREEDOM) clinical trial, data demonstrated that denosumab was associated with a statistically significant increase in serious infection events (SAEs), specifically cellulitis and erysipelas (12) . Therefore, denosumab may be a two-edged sword for cancer patients threatened by COVID-19. Due to the failure of the immune system and weakened autoreactive responses, patients may be more susceptible to COVID-19 infections. In a recent survey, experts even raised concerns that the risk of infection could be worsened if RANKL inhibitors are concomitantly employed with other biological agents of anti-TNF (13). Therefore, clinicians and researchers should especially be concerned about the mutual effects of vaccination and denosumab treatment. However, studies that focus on the efficacy and safety of the vaccine for patients with spinal tumors treated with denosumab have not been conducted as yet.

In our study, we aimed to assess the efficacy and safety of the COVID-19 vaccine for patients with spinal tumors undergoing denosumab treatment. The aim of our study is to provide novel insights for the prevention of COVID-19.

## 2 Materials and methods

### 2.1 Study design

We conducted a retrospective study at the Shanghai ChangZheng Hospital on the further investigation of the influence between COVID-19 vaccines and denosumab from January 22^nd,^ 2021 to June 1^st,^ 2021. Our research investigators also shared a questionnaire through social media channels, including WeChat and Weibo, to obtain more information. Unsolicited clinically unidentified signs and symptoms were also recorded when a patient spontaneously volunteered to report symptoms during consultation. Our research was also approved by the local Ethics Committee.

### 2.2 Patients

All participants enrolled in this study had to meet the following inclusion criteria: (1) the spinal sites were pathologically diagnosed as metastatic malignancy or primary spinal tumor during the period starting January 1^st,^ 2019 to January 22^nd,^ 2021; (2) the patients were treated using 120 mg of denosumab per month (3) over eighteen years of age. (4) participants were able to understand basic details regarding the disease. The exclusion criteria for participation included: (1) a history of COVID-19; (2) treatment using immunosuppressive medication based on programmed cell death 1 (PD-1), hormone therapies such as prednisone, or methylprednisolone concerning brakes in immune tolerance mechanisms, patients with bone marrow suppression caused by targeted therapy and aggressive chemotherapy; and (3) patients who refused to provide consent.

### 2.3 Vaccination

At present, two types of vaccines, mRNA vaccines, and inactivated vaccines have been widely used in our research. The inactivated vaccine, CoronaVac (Sinovac Life Sciences, Beijing, China), was created using African green monkey kidney cells (Vero cells) that have been inoculated with SARS-CoV-2 (CN02 strain). The other COVID-19 vaccine, which is a replicate of a defective Ad5 vectored vaccine, was also developed by the Beijing Institute of Biotechnology (Beijing, China) and CanSino Biologics (Tianjin, China). It was constructed by cloning an optimized full-length spike gene based on Wuhan-Hu-1 (GenBank accession number YP_009724390) of the tissue plasminogen activator signal peptide gene into an E1 and E3 deleted Ad5 vector (14). As reflected in the survey, all the mRNA vaccine was only injected for a single dose (5ml) (14). All the inactivated vaccines were planned to be injected at 0-14 day, and 0-28 day for 5μg each time (15).

### 2.4 Efficacy and safety

Data on the basic characteristics of the patients, including demographic characteristics such as age, sex, Body Mass Index (BMI), pathological diagnosis, and comorbidities with rheumatic disease, were recorded. Venous blood was tested regularly on day 20 following denosumab treatment and at 6 months after vaccination. The efficacy of the vaccine was determined by the positive state of anti-SARS-Cov-2 immunoglobulin G (IgG) antibodies (16) (IgG antibodies were tested using the domain of the spike protein of the virus used in a commercial enzyme-linked immunosorbent assay (ELISA) kit as recommended by the manufacturer at local hospitals)

Safety was evaluated using weekly medical consultations to identify treatment-related adverse events resulting from both the vaccine for patients treated by denosumab. Related adverse events were classified into 4 levels according to the National Cancer Institute Common Terminology Criteria for Adverse Events (17): Grade 1 (mild; does not interfere with activity); Grade 2 (moderate; interferes with activity), Grade 3 (severe; prevents daily activity), and Grade 4 (potentially life-threatening; required emergency department visit or admission to hospital).

### 2.5 Statistical analysis

Categorical variables are presented as frequency and percentage. Continuous variables are presented as a pie chart to determine distribution. χ2 test and Fisher Exact Test were used to compare between the groups. Due to the sample size and concerns regarding false positives in the multivariate analysis, the subgroup analyses were not conducted on different types of cancer (18). All statistical tests were two-tailed. A p< 0.05 was regarded as statistically significant. We also used minimal necessary adjustments on the covariates using directed acyclic graphs (DAG) (**Figure S1** in Appendix A).

## 3 Results

### 3.1 Patients

A total of four hundred cancer patients with primary or metastatic spinal tumors undergoing denosumab treatment were included. 233 patients were administered both the COVID-19 vaccine and denosumab, while 167 patients who were treated with denosumab without being administered the vaccine served as the control cohort. The vaccine group was composed of 124 men (53.22%) and the control group comprised 89 men (53.29%).

**Figure 1:**
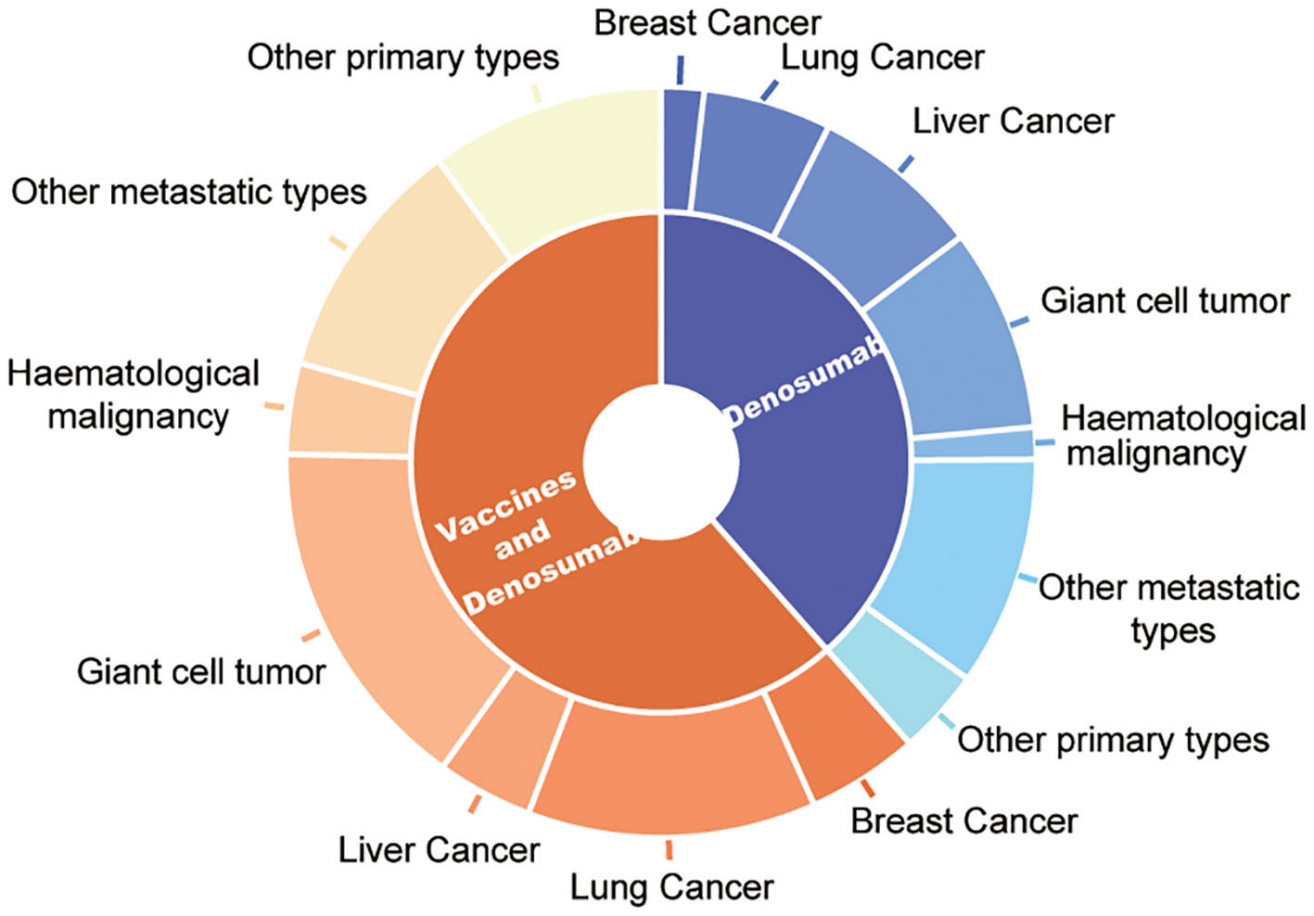
Oncological characteristic distribution of patients treated by denosumab and vaccine and only denosumab therapy.

Among these patients, the most common type of cancer was lung cancer (n=42;18.03%) followed by breast (n=21; 7.87%), liver (n=24; 6.74%), and other metastatic cancers (n=24; 10.30%). Among the 111 (47.64%) patients with primary osseous spinal tumors included in the vaccine group, 67 (25.09%) patients had giant cell tumor of the bone, 26 (11.16%) patients had osteosarcoma, 8 (3.43%) patients had fibrous dysplasia, while 10 (4.29%) patients had chondrosarcoma. A similar oncological distribution was observed in the control group. The types of cancer included in the control cohort were 38 (22.75%) patients with lung cancer, 32 (19.16%) patients with liver cancer, 29 (17.37) patients with metastatic cancers in other tissues, 38 (22.75%) patients with giant cell tumor of the bone, 10 (5.99%) osteosarcoma patients, 2 (1.20%) fibrous dysplasia patients, and 4 (2.40%) patients with chondrosarcoma in the osseous spinal tumors (**Table 1** and **Figure 1**). Furthermore, comorbidities of the patients did also not show any statistical differences, such as hypertension (70, 30.04%; 45, 26.95%), diabetes (63, 27.04%; 45, 26.95%), and other conditions (29, 12.45%; 26, 15.57%).

**Table 1:** Oncological characteristics.

Characteristics	Treated by vaccine and denosumab	Treated by denosumab	P Value
Number (n)	233(58.25%)	167(41.75%)	
Age, mean (SD)	48.37 (15.77)	56.31(15.50)	0.000
Male [%]	124(53.22%)	89(53.29%)	0.988
BMI (SD)	19.86(3.50)	20.31(3.84%	0.216
Respiratory disorders, no. (%)	0	0	–
COPD, no. (%)	20(8.58%)	16(12.03%)	0.731
Diabetes, no. (%)	63(27.04%)	45(26.95%)	0.984
Hypertension, no. (%)	70(30.04%)	50(29.94%)	0.741
Heart disease, no. (%)	6(2.58%)	9(5.39%)	0.144
Stroke, no. (%)	3(1.29%)	1(0.60)	
Autoimmune conditions, n(%)			
Systemic lupus erythematosus	7	4	
Chronic viral infections	27	26	
Oncological Characteristics			
Type of Cancer, n(%)			
Primary osseous spinal tumors	111(47.64%)	54(32.34%)	
Giant cell tumor of bone	67(25.09%)	38(22.75%)	
osteosarcoma	26(11.16%)	10 (5.99%)	
chondrosarcoma	10(4.29%)	4(2.40%)	
fibrous dysplasia	8 (3.43%)	2 (1.20%)	
spinal metastatic carcinoma	122(52.36%)	113(67.66%)	
Lung	42(18.03%)	38(22.75)	0.028
Breast	21(7.87%)	8(4.79)	0.108
Liver	18(6.74%)	32(19.16)	0.001
haematological malignancy	17(6.37%)	6(3.59)	0.117
Other metastatic types	24(10.30%)	29 (17.37)	0.040

Denosumab and Vaccine and Denosumab therapy.

### 3.2 Efficacy of COVID-19 vaccines in spinal tumor patients

In the vaccinated cohort, 209 89.70% of patients received the inactivated vaccine, while the other 24 patients received the mRNA vaccine. All patients were tested after being vaccinated and the results showed that 147 (83.52%) of these patients showed positive serology after examination for the presence of antibodies. Among the patients who were positive for antibodies, 162 (69.52%) patients had received one dose of the inactivated vaccine, while 47(20.17%) had received two doses of the inactivated vaccine. Over the study period, 88 individuals (81.48%) who were injected with one inactivated vaccine showed effective antibodies against SARS-CoV-2. The results in the 41 patients who had received the second vaccine showed an 87.23% antibody response in the survey. Only one dose of the mRNA vaccine was administered to 24 patients treated with denosumab. Among them, 18 individuals (85.71%) who received the mRNA vaccine also showed a seropositive serologic status (**Table 2**). In the univariate analysis, all potential risk variables were listed and showed to have a statistically significant influence on the immune response to the vaccine (p<0.005). The risk factors included age, sex, BMI, comorbidities, and the type of vaccine. The results of the multivariable logistic regression (**Table 3**) did not indicate the causality of these negative seroconversions and potential factors.

**Table 2:** Efficacy of serologic response in patients treated by denosumab and vaccine.

Patients	No. of test (ratio of test to injected patients)	No. of Positive (ratio of positive to test patients)	P value
Vaccinated			
Inactive vaccine			
One dose	108(66.67%)	88(81.48%)	0.000
Two dose	47(100%)	41(87.23%)	0.000
mRNA vaccine	21(87.5%)	18(85.71% )	0.000
Primary spinal tumors	85 (76.58%)	73(49.66%)	0.000
Metastatic spinal tumors	91 (74.59%)	73(50.34%)	0.000

**Table 3:** Univariable and multivariable analysis of factors potentially associated with serologic response.

Characteristics	Seropositive serologic status percentage	Univariable analysis P Value	Multivariable analysis OR (95% CI)	P Value *
Age > 50 (ref: Age ≤ 50)	41.50% (61/147)	0.000	0.972(0.936-1.009)	0.138
Male (ref: female)	50.34% (74/147)	0.002	0.623(0.263-1.475)	0.282
BMI>24 (ref: BMI≤24)	12.93% (19/147)	0.000	0.949(0.846-1.066)	0.378
Comorbidities (ref: no comorbidities)	18.37% (27/147)	0.000	0.921(0.324-2.621)	0.878
Metastatic tumor	49.66% (73/147)	0.000	0.923(0.418-2.620)	0.923
Primary tumor	50.34% (74/147)			
Type of Vaccines				0.792
Inactivated Vaccine				
One shoot	81.48% (88/105)	0.000		0.643
Two shoots	87.23% (41/47)	0.000		0.579
mRNA Vaccine	85.71% (18/27)	0.000		

*p< 0.05 is considered statistically significant.

### 3.3 Safety of COVID-19 vaccines for spinal tumor patients

**Figure 2:**
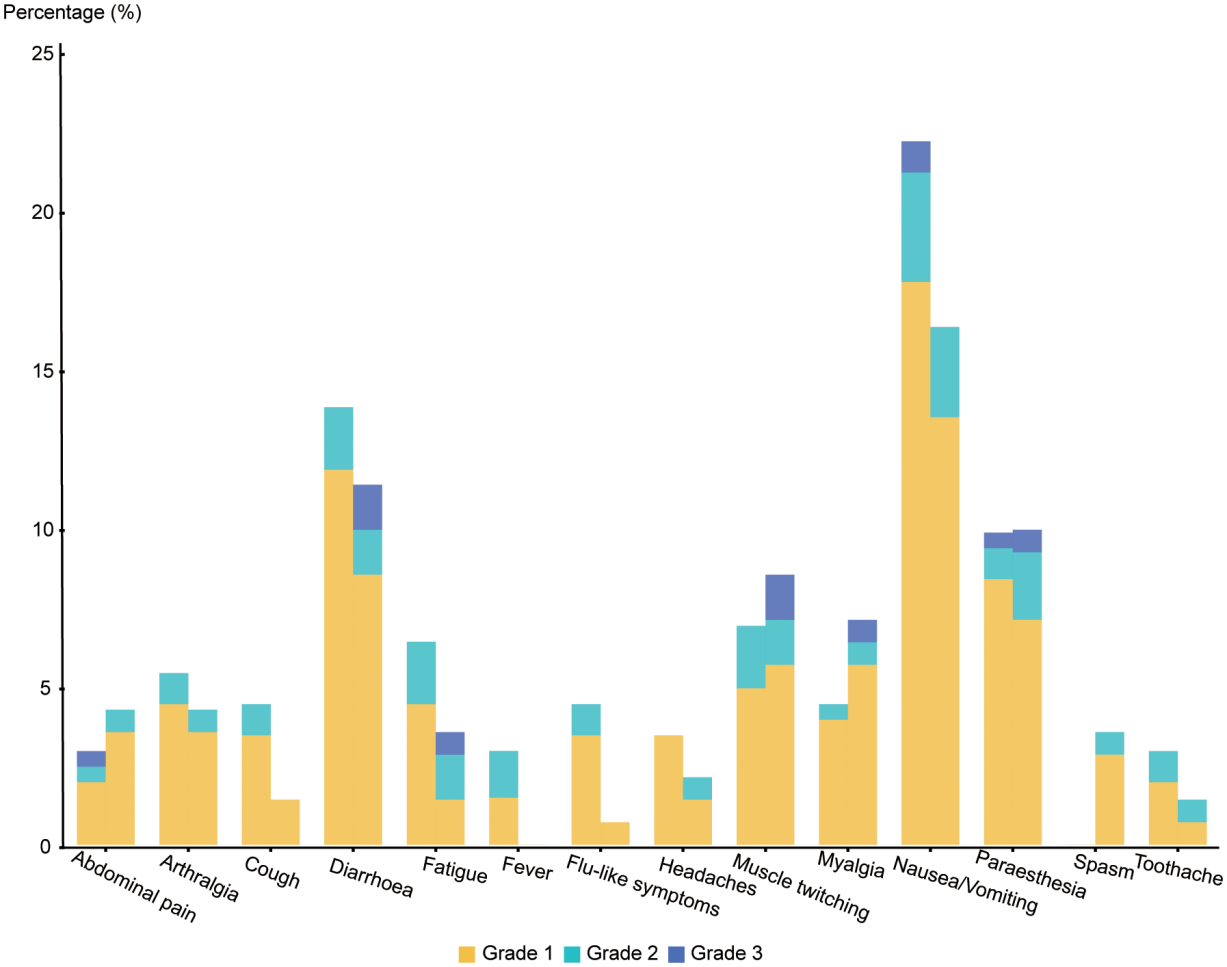
Related systemic adverse events between two cohorts.

**Figure 3:**
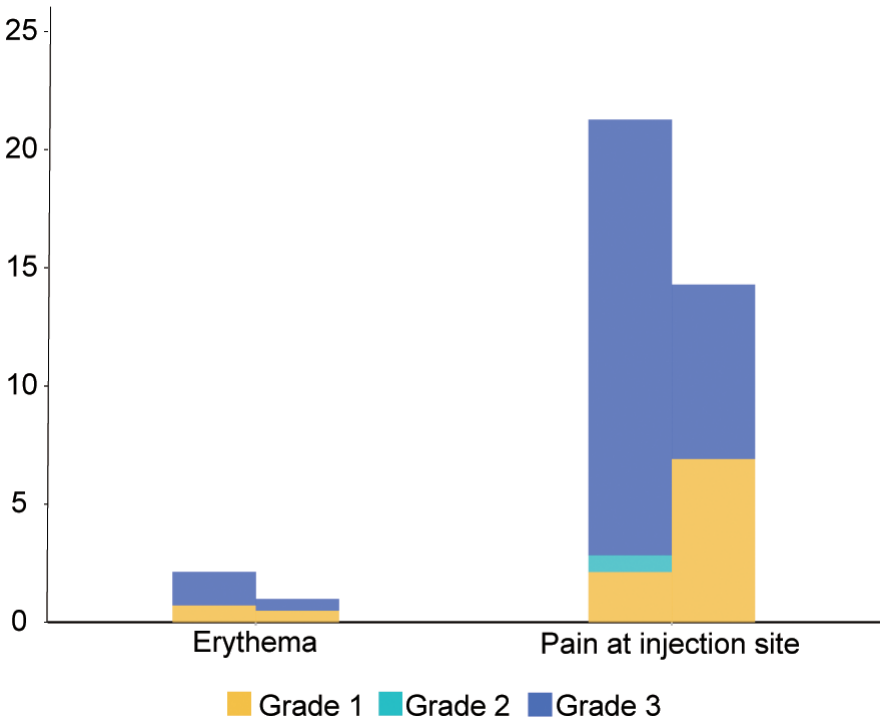
Related local adverse events between two cohorts.

Our research showed that no fatal super sensitivity or life-threatening responses occurred in either group regardless of whether the patients were vaccinated or not. Among the 400 patients, it was observed that systemic adverse events were more frequent in the vaccinated group than in the placebo group (170 events (83.74%) vs. 99 events (70.21%)) (**Table 4** and **Figure 2**). The most common systemic adverse event following vaccination was nausea or vomiting (45 events, 22.17% vs 23events, 16.33%), with most events distributed in grade 1 (36 events, 17.74% vs 19 events, 13.48%) and grade 2 (7 events, 3.45% vs 4 events, 2.84%) tumors. The systemic adverse events included nausea or vomiting, fatigue, headaches, muscle or joint pain, fever, gastrointestinal complications, and flu-like symptoms, which showed no significant differences between the two cohorts. Interestingly, statistically fewer participants in the vaccine group recorded local adverse events than in the placebo group (16 events, 7.88% vs 33 events, 23.40%), including pain at the injection site (15 events, 7.39% vs 30 events, 21.28%) and Erythema (1 event, 0.49% vs 3 events, 2.13%) (**Figure 3**). All adverse events recorded following vaccination were characterized as short-term, mild-to-moderate adverse events. None of the reported complications required admission to the hospital or further special interventions.

**Table 4:** Local and systemic adverse events reported after injection of the COVID-19 vaccine and denosumab.

Adverse event	Treated by vaccine and denosumab				Treated by denosumab					OR	P value
	Any Grade	Grade 1	Grade 2	Grade 3	Any Grade	Grade 1	Grade 2		Grade 3		
Local adverse events	16(7.88%)				33(23.40)					0.337 (0.193-0.588)	0.000^*^
Pain at injection site	15(7.39)	14(6.90)	1(0.49)	0	30(21.28)	26(18.44)	3(2.12)		1(0.71)	0.347 (0.194-0.621)	0.000^*^
Erythema	1(0.49)	1(0.49)	0	0	3(2.13)	2(1.42)	1(0.71)		0	2.232 (0.024-2.203)	0.379
Systemic adverse events	170(83.74)				99(70.21)					1.193 (10.54-1.349)	0.003^*^
Fever *	3(1.48)	3(1.48)	0	0	0	0	0		0	–	0.390
Flu-like symptoms	9(4.43)	7(3.45)	2(0.98)	0	1(0.71)	1(0.71)	0		0	6.251 (0.801-48.792)	0.090
Fatigue	13(6.40)	9(4.43)	4(1.97)	0	5(3.55)	2(1.42)	2(1.42)		1(0.71)	1.806 (0.648-5.343)	0.242
Toothache	6(2.96)	4(1.97)	2(0.98)	0	2(1.42)	1(0.71)	1(0.71)		0	2.084 (0.427-10.175)	0.571
Cough	9(4.43)	7(3.45)	2(0.98)	0	2(1.42)	2(1.42)	0		0	3.126 (0.686-14.249)	0.211
Headaches	7(3.45)	7(3.45)	0	0	3(2.13)	2(1.42)	1(0.71)		0	1.621 (0.426-6.161)	0.696
Arthralgia	11(5.42)	9(4.43)	2(0.98)	0	6(4.26)	5(3.55)	1(0.71)		0	1.273 (0.482-3.363)	0.813
Myalgia	9(4.43)	8(3.94)	1(0.49)	0	10(7.09)	8(5.67)	1(0.71)		1(0.71)	0.625 (0.261-1.499)	0.411
Diarrhea	28(13.79)	24(11.82)	4(1.97)	0	16(11.36)	12(8.52)	2(1.42)		2(1.42)	1.216 (0.684-2.161)	0.504
Nausea/Vomiting	45(22.17)	36 (17.74)	7(3.45)	2(0.98)	23(16.33)	19(13.48)	4(2.84)		0	1.238 (0.779-1.968)	0.362
Abdominal pain	6(2.96)	4(1.97)	1(0.49)	1(0.49)	6(4.26)	5(3.55)	1(0.71)		0	1.158 (0.431-3.113)	0.771
Muscle twitching	14(6.90)	10(4.93)	4(1.97)	0	12(8.51)	8(5.67)	2(1.42)		2(1.42)	0.810 (0.386-1.699)	0.578
Paraesthesia /Tingling	20(9.85)	17(8.37)	2(0.98)	1(0.49)	14(9.93)	10(7.09)	3(2.13)		1(0.71)	0.992 (0.519-1.897)	0.981
Spasm	0	0	0	0	5(3.55)	4(2.84)	1(0.71)		0	–	0.025
Other adverse events	7(3.45)	0	0	0	3(2.13)						
Chest pain	0	0	0	0	0	0	0		0	–	–
Dyspnoea	0	0	0	0	0	0	0		0	–	–
Hypotension	0	0	0	0	0	0	0		0	–	–
Pruritus	7(3.45)	6(2.96)	1(0.49)	0	3(2.13)	2(1.42)	1(0.71)		0		
Total	203	168	33	2	141	107	24		8		

*Defined as subjective self-reported fever symptoms by patients. Those who did not have a recorded temperature either using a home thermometer or during clinical assessment were categorised as grade 1 fever.

All the participants were analyzed for their risk of developing related adverse events, serious adverse events (>2 grades), and any grade of systemic adverse events. Cancer patients with fewer comorbidities and metastatic sites were less likely to report adverse events of any grade compared with those with more comorbidities and metastatic sites (OR 2.520 [95%CI 1.430–4.442]; p = 0.001 vs. OR 1.839 [95%CI 1.128–2.996]; p = 0.015). However, age, BMI, comorbidities, metastatic cancer, or receiving the vaccines, did not show any statistical difference in developing grade ≥2 adverse events (**Tables 4**, **5** and **Supplementary Data**).

**Table 5:** Risk of total any grade adverse events (n = 400).

Risk Factors	Adjusted OR	95%CI (Lower)	95%CI (Upper)	p-Value
Age	0.979	0.979	0.963	0.12
Male (ref: female)	0.724	0.482	1.086	0.118
BMI	1.001	0.949	1.056	0.972
Comorbidities (ref: no comorbidities)	2.520	1.430	4.442	0.001*
Metastatic cancer (ref: non-metastatic cancer or only skeletal system cancer)	1.839	1.128	2.996	0.015
Vaccine (ref: receiving non-any kind of vaccine)	0.784	0.503	1.220	0.280

Treated by denosumab. *p< 0.05 is considered statistically significant.

## 4 Discussion

The spine is one of the most common sites of metastatic and primary osseous tumors (19, 20). All active untreated spinal lesions can lead to local irreversible events that are associated with spinal cord compression and pathological fractures (21). Once these events occur, they have a devastating destructive impact on the quality of life and require further multidisciplinary synthetic therapy. The nuclear kappa B ligand (RANKL) signaling pathway has been proven to be highly expressed in osteoclasts and performs an important role in the regulation of bone resorption–production. Due to the inhibition of osteoclast-mediated bone destruction, denosumab has been approved by the FDA for the treatment of unresectable giant cell tumor of bone and metastatic spinal cancers (22). However, a study on RANKL expression in primary osseous spinal tumors showed that RANKL is also expressed at high levels in fibrous dysplasia, osteosarcoma, chondrosarcoma, and enchondroma (23). Animal experiments and drug sensitivity tests have also shown that denosumab can inhibit the growth of primary tumors (24). Based on the results of preclinical research studies, denosumab has been gradually applied as a novel method of treatment for primary spinal tumors (23, 25, 26). Therefore, patients with metastatic malignancies and primary spinal tumors being treated with denosumab were included and analyzed in our research study. Nonetheless, the global outbreak caused by COVID-19 has become an unparalleled challenge, especially for the comprehensive treatment of patients with spinal tumors since December 2019 (1, 27). It has been reported that severe complications were more likely to occur in elderly and fatal patients who have been diagnosed with malignant tumors (28). Therefore, vaccination against severe acute respiratory syndrome-related coronavirus-2 (SARS-CoV-2) may represent an unprecedented method of treatment in the fight against the pandemic. However, only a few clinical studies have focused on the unique responses of patients with spinal tumors to COVID-19 vaccines. According to our knowledge, this is the first research study to determine the efficacy of the inactivated and mRNA COVID-19 vaccines for patients being treated using denosumab. Therefore, our study aimed to demonstrate whether COVID-19 vaccination has an influence on the rate of adverse events and the efficacy of treatment in this group of patients.

A positive result for antibodies and significant IgG levels are potential predictive factors of vaccine resistibility and efficacy (29). Our results indicated that 81.48% and 85.71% of patients who were administered one dose of the inactivated vaccine and the mRNA vaccine, respectively, showed a positive serological response against COVID-19. After the second inactivated vaccine was injected, the rate of seroconversion increased from 81.48% to 87.23%. The interim analysis of the results of 2 randomized clinical trials confirmed 95.8% efficacy of the inactivated vaccine against SARS-CoV-2 in healthy adults (15). Another research study conducted on 364 cancer patients who received the inactivated vaccine showed that the rate of seroconversion in cancer patients with breast cancer may even reach 93.3%, and may be as high as 94.7% in patients with digestive cancers (30). Our investigation showed that vaccinated patients treated with denosumab seroconverted antibodies were at levels less than that of normal individuals and cancer patients. Furthermore, early clinical data appeared to show a higher tendency of COVID-19 infection among patients being treated with denosumab (31). Clinical trials have also suggested that denosumab therapy may increase the risk of infection in cancer patients. Severe infections were reported at a rate of 2.3% vs 0.8% in denosumab-treated patients compared with placebo-treated patients with early-stage breast cancer (32). Similar results were also obtained in androgen-dependent prostate cancer patients (33). Compared with the results of clinical trials and studies on the molecular mechanisms involved (34–36), a lower vaccination efficacy and higher risk of infection may be associated with immunity and denosumab treatment. The potential influence of immunosuppression on RANKL inhibitors may be because RANKL expression and the immune system share multiple pathways involved in B-cell differentiation and T-cell survival (37). Our results showed that although a lower rate of seroconversion was observed in the vaccine group, a sufficient antibody response to SARS-CoV-2 is acceptable for patients with spinal tumors. We also evaluated whether denosumab treatment has an influence on vaccination efficacy. In the multivariate analysis, the type of vaccine, age, sex, BMI, and the metastatic site did not lead to a significant difference while the potential risk factors in the univariate analysis were proven to be correlated. Therefore, the relationship between vaccine immunogenicity and denosumab treatment may need to be urgently further investigated.

Comparison with the placebo group showed a higher tendency of abdominal adverse events in the cohort of patients with cancer who were vaccinated but the result was not statistically significant. The rate of nausea or vomiting and diarrhea reached 22.17% and 13.79%, respectively, and were the most common systemic adverse events reported in our cohort. However, in post-COVID-19 vaccine trial conducted on cancer patients vaccinated (38, 39) with the inactivated vaccine, fatigue and headaches were the most frequently reported systemic adverse events (8.17% and 8.79%, respectively) but only reached a rate of 6.40% and 3.45%, respectively, in our research study. Interestingly, our results suggest that patients who have been vaccinated may have a lower rate of local adverse events than the control group. Moreover, in our research study, the rate of local adverse events, including pain and erythema, was much lower than that which was previously reported in studies conducted on elderly patients (pain 42.4% and erythema 1.7%) (40) and cancer patients (pain 31.52% and erythema 33.46%) (39). Due to the method of vaccine administration, patients being treated with denosumab experience more pain as a result of both subcutaneous injections of the vaccine and disease treatment. Additionally, it can be observed that local adverse events are more subjective and are usually neglected compared to systemic adverse events.

Denosumab treatment may also lead to complications, such as fatigue, nausea, dyspnea, and hypocalcemia (41). Therefore, we aimed to determine whether denosumab treatment could influence the rate of any grade of adverse events caused by crosstalk with the vaccine. Fever and flu-like symptoms are also frequently reported complications of denosumab, but the rate of these symptoms did not increase in the cohort that was vaccinated and treated with denosumab compared with vaccinated cancer patients who were not treated with denosumab (39, 42, 43). Osteonecrosis of the Jaw (ONJ) is a previously reported complication of denosumab that affects 8% of therapeutic patients. Nevertheless, we did not observe the occurrence of ONJ in our research study. The incidence of toothache in cancer patients who were vaccinated and treated with denosumab was similar to that of patients who were being treated with denosumab but not vaccinated (2.96% vs 1.42%). As Raje et al (44) have indicated, the higher incidence may be related to prolonged exposure to therapy during the trial, which might account for the absence of ONJ incidence in our study.

We also analyzed the risk of the occurrence of adverse events in patients being treated with denosumab who were vaccinated. Analyses based on age, sex, and the metastatic site did not show significant differences in the patient cohorts, while comorbidities represented a risk factor for developing a mild or severe grade of adverse events and systemic adverse events. The application of vaccination in the current study suggests the relative safety of the vaccine, as vaccination was not a risk factor for adverse events.

A total of 122 (52.36%) patients were diagnosed with metastatic cancer in the vaccine group and some of those patients had been treated using other types of chemotherapy or molecular targeted therapies for cancer *in situ* and had other potential metastatic sites. Up till now, the specific relationship between chemotherapy and virus infection has not been verified (45, 46). Even if immunotherapy was excluded, we cannot demonstrate that chemoradiotherapy or multiple metastatic sites will not obstruct immunological functions or the seroconversion of antibodies. However, the multivariate analysis showed that there were no statically significant differences observed between primary tumors and metastatic cancer. This finding may indicate that chemoradiotherapy or multiple metastatic sites are not major factors that affect the efficacy and safety of the vaccine. However, further research should also be conducted to obtain more persuasive evidence on the effect of vaccination and other anticancer treatments.

Our study also has serval limitations. The clinical information was retrospectively collected. The choice of type and dose of COVID-19 vaccine depended more on limited supply, vaccine acceptancy, culture, and sociodemographic factors instead of clinical intervention. A study conducted on a larger cohort along with a longer follow-up period should be conducted to fully assess the effects of vaccination and denosumab treatment. Our results confirmed a satisfactory rate of seroconversion after vaccination in patients with spinal tumors being treated with denosumab. Serve adverse events were not observed in our study. Therefore, at this historically challenging moment, our results indicate that COVID-19 vaccines are likely to be safe and effective for patients with spinal tumors being treated using denosumab. Based on the results of this study we recommend that spinal tumor patients being treated using denosumab should get vaccinated in a timely manner to get through current pandemic conditions.

## Data availability statement

The original contributions presented in the study are included in the article/**Supplementary Material**. Further inquiries can be directed to the corresponding authors.

## Ethics statement

This study was approved by the Ethics Committee of Chang Zheng hospital and granted a waiver of informed consent from study participants.

## Author contributions

PW, BL, and SZ contributed to conception and design of the study. YX organized and collected the database. ZZ performed the statistical analysis. PW wrote the first draft of the manuscript. SD, DB, and HY wrote sections of the manuscript. WX and JX supervised the whole project. All authors contributed to the article and approved the submitted version.
