# Supplementary figures and images for: Efficacy and safety of COVID-19 vaccines for patients with spinal tumors receiving denosumab treatment: An initial real−clinical experience study
Source: Front Oncol. 2023 Mar 22;13:1034466. doi: 10.3389/fonc.2023.1034466 (PMC10073434; doi:10.3389/fonc.2023.1034466)

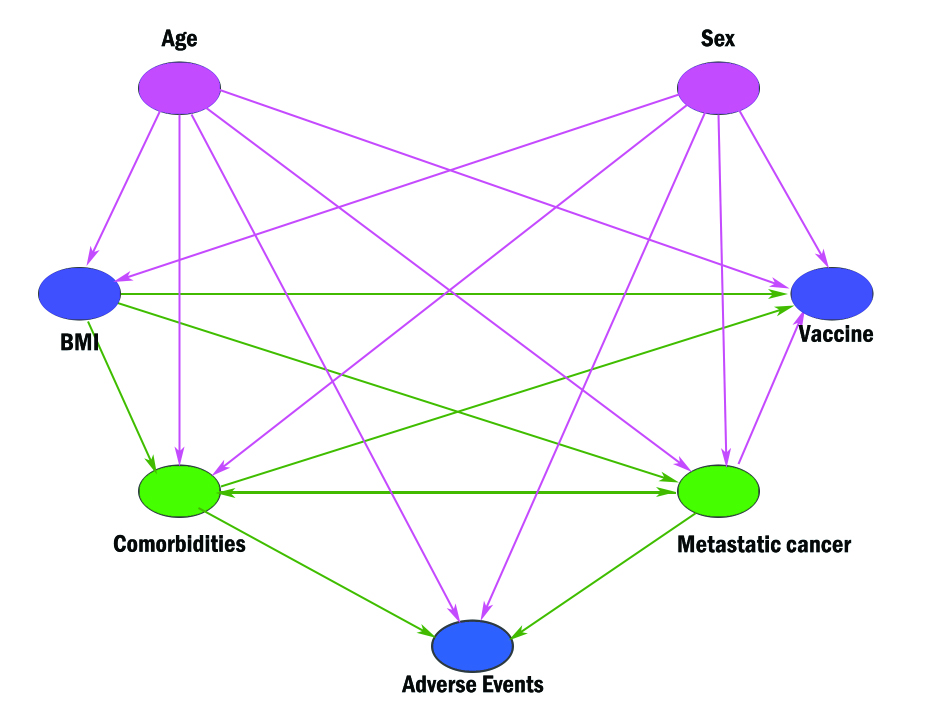

Supplement: Supplementary file 1 [file Image_1.jpeg]
